# Treatment patterns in patients with systemic lupus erythematosus in New Zealand

**DOI:** 10.1177/09612033241274911

**Published:** 2024-08-16

**Authors:** Chunhuan Lao, Philippa Van Dantzig, Nikki Tugnet, Ross Lawrenson, Douglas White

**Affiliations:** 1 Medical Research Centre, 3717 The University of Waikato, Hamilton, New Zealand; 2 Rheumatology Department, 3718 Waikato Hospital, Hamilton, New Zealand; 3 Faculty of Medical and Health Sciences, 1415 University of Auckland, Auckland, New Zealand; 4 Waikato Clinical School, 1415 University of Auckland, Auckland, New Zealand

**Keywords:** Systemic lupus erythematosus, lupus, mortality, underlying cause of death, ethnic difference

## Abstract

**Objectives:**

This study aims to explore the treatment pattern of systemic lupus erythematosus (SLE) in Aotearoa/New Zealand.

**Methods:**

SLE patients were linked to the pharmaceutical dispensing data. The use of publicly funded anti-malarials, immunomodulators, biologics, glucocorticoids and bisphosphonates were compared by gender, ethnicity, age group, socioeconomic status and year of SLE identification. Adherence to hydroxychloroquine was examined using the medication possession ratio (MPR), with a MPR of ≥0.8 considered as high adherence.

**Results:**

Of the 2631 SLE patients, 73.8% used hydroxychloroquine, 64.1% used immunomodulators/biologics and 68.0% used 5 mg or more prednisone daily for at least 90 days. Women were more likely to use hydroxychloroquine than men. Asian patients had a different treatment pattern than other ethnic groups, and Māori were less likely to use hydroxychloroquine. The proportions of patients using different treatments decreased with age. Of the patients using hydroxychloroquine, 54.5% had high adherence. For patients over 40 years old and on long term prednisone, 47.3% had bisphosphonates and this figure was 17.8% for patients under the age of 40 years old. Patients with better socioeconomic status had a higher probability of using bisphosphonates than patients with lower socioeconomic status.

**Conclusions:**

Adherence to hydroxychloroquine in these patients varied and was lower in men and in Māori. Prednisone is commonly prescribed and used long term. Half of those over the age of 40 years old co-administered bisphosphonate. Further research is needed to identify the reasons for these discrepancies on SLE treatments by gender, ethnicity, age and socioeconomic status.

## Introduction

Systemic Lupus Erythematosus (SLE) is a multi-system autoimmune disease presenting with a constellation of clinical and laboratory features. It is characterised by the presence of anti-nuclear antibodies (ANA) and has a variable prognosis with disease severity ranging from mild to life-threatening. The treatment of SLE focuses on achieving remission or low disease activity, preventing damage accrual from inflammation and improving quality of life. It is important to minimise medication side effects whilst treating the disease.^1,2^ Treatment includes non-pharmacologic management (i.e. smoking cessation, reduction of ultraviolet light (UV) exposure) and pharmacological measures. Anti-malarial medications are recommended for all patients with SLE and hydroxychloroquine is the most commonly used medication. They have multiple benefits in SLE including preventing flares, increasing long term survival and preventing irreversible organ damage.^3^ Limitations to the use of hydroxychloroquine include skin and gastrointestinal intolerance, cardiac toxicity and retinopathy with long term use. These side effects may lead to low adherence to hydroxychloroquine. Previous studies have demonstrated that 38%–85% of patients had low adherence to hydroxychloroquine.^4–6^

When managing patients with SLE, treatment depends on the organs involved, severity of disease, patient characteristics (i.e. planning pregnancy, comorbidities), safety and cost.^1,2^ Glucocorticoids are used for rapid disease control but have detrimental effects, including irreversible organ damage, as well as deleterious effects on bone, weight, skin, eyes and adrenals.^1,7^ These can be used in a topical, oral or intravenous form. Immunomodulators facilitate glucocorticoid tapering and may prevent disease flares.^8^ Conventional immunomodulators used for SLE include methotrexate, azathioprine, mycophenolate mofetil, cyclophosphamide, tacrolimus and ciclosporin.

The development of biologic medications for the treatment of SLE has lagged behind other rheumatic diseases. B-cell targeting agents that have been approved by the Food and Drug Administration (FDA) include Belimumab and Rituximab. Rituximab has been funded for the treatment of SLE in Aotearoa/New Zealand since 2014, whereas Belimumab is not yet available. In 2021, anifrolumab and voclosporin were FDA-approved and are routinely used in the management of SLE or lupus nephritis internationally. Both agents are yet to be funded in New Zealand. There are trials ongoing for medications targeting various pathways of inflammation in SLE, including for obinituzumab (antiCD20) and deucravacitinib (tyrosine kinase inhibitor)

European League against Rheumatism (EULAR) and the American College of Rheumatology (ACR) have published guidelines for the management of SLE.^1,2^ Treatment regime, duration of treatment, route of administration and dosage of medication are individualized depending on the symptoms, organ involvement, disease severity and availability of medications.^9^ Previous studies have shown that the management of SLE varies with ethnicity, age at diagnosis, and specialisation of the treating physician.^10–13^ The treatment patterns of SLE and the factors that affect the management of SLE have not been reported in Aotearoa/New Zealand. This study aims to fill this gap and to explore the treatment patterns of SLE in Aotearoa/New Zealand.

## Methods

Patients with SLE were identified through searching the National Minimum Dataset (NMDS) and the Mortality Collection using the ICD-10 code “M32” and searching the Death Certificates using the keyword “systemic lupus erythematosus”. The date of first identification for SLE was determined as the earliest occurrence of an inpatient event with the ICD-10 code “M32” in the NMDS or the first date from the National Non-admitted Patient Collection (NNAPC) for an outpatient event in the Rheumatology department or Renal Service. SLE cases were linked with the Pharmaceutical Collection (PHARMS) using the patients’ National Health Index (NHI) numbers, unique identifiers for individuals utilising health and disability services in Aotearoa/New Zealand. The NMDS documented inpatient and day patient events, and the NNAPC captured outpatient events and emergency department occurrences. The NMDS and NNAPC cover all public hospitals and over 90% of private hospitals. The Mortality Collection included date and cause of death data coded in ICD-10, while Death Certificates provided more current death records not yet incorporated into the Mortality Collection. The PHARMS dataset contained claim and payment information from pharmacists for subsidised dispensing. Details of data validation and patient characteristics of these SLE patients have been published.^14,15^ The study period was from January 1, 2005 when the electronic records of pharmaceutical dispensing was first available, to December 31, 2021 which was the last follow-up date for this study. Only patients with 1 year or more of follow-up were included in this study.

The use of publicly-funded anti-malarials (hydroxychloroquine), immunomodulators (mycophenolate, methotrexate, leflunomide, azathioprine, cyclophosphamide, tacrolimus and ciclosporin), biologics (rituximab), glucocorticoids (prednisone, methylprednisolone and prednisolone) and bisphosphonates (alendronate sodium, zoledronic acid and risedronate) for SLE patients were investigated. The treatment pattern was compared by gender, ethnicity, age group, socioeconomic status and year of first identification of SLE. Differences by subgroup were examined with Pearson’s chi-square test. Use of self-funded medications or medications obtained in clinical trial or by compassionate supply including Belimumab was not included in this study. Ethnicity was classified into Māori, Pacific, Asian and European/Other ethnic groups. Ethnicity is self-identified in New Zealand. Age was stratified into eight groups: less than 20, 20-29, 30-39, 40-49, 50-59, 60-69, 70-79 and 80 years or older. Socioeconomic deprivation was defined using the New Zealand Index of Deprivation 2018 (NZDep 2018) analysed as quintile, from 1 (least deprived) to 5 (most deprived).^16^ Year of first identification of SLE was categorised into five groups: (1) before 2010, (2) 2010-2012, (3) 2013-2015, (4) 2016-2018, and (5) 2019-2021. The proportion of patients treated with prednisone for 5 mg or more daily for at least 90 days and the proportion of patients treated with bisphosphonates were calculated.

Adherence to hydroxychloroquine from the first dispensing date to the last follow-up date was examined using the medication possession ratio (MPR). MPR was defined as the proportion of days that a patient has medication cover, with an MPR of 0.8 or higher (i.e. cover for at least 80% of days) indicating ‘high’ level of adherence, and an MPR of less than 0.8 indicating ‘low’ level of adherence. Hydroxychloroquine is dosed at 200-400 mg daily based on patient weight. We did not have data on patient weight, therefore the adherence to hydroxychloroquine was analysed under the assumption that patients were dosed at 200 mg daily. Adherence to hydroxychloroquine was investigated by gender, ethnicity, age group, socioeconomic status and year of first identification of SLE.

Odds ratios of using hydroxychloroquine, immunomodulators/biologics, prednisone for 5 mg or more daily for at least 90 days, high adherence to hydroxychloroquine and bisphosphonate therapy for Māori, Pacific and Asian patients compared with European/Others were estimated by binary logistic regression, after adjustment for gender, age group, socioeconomic status and year of first identification of SLE. The 95% Confidence interval (CI) of the adjusted odds ratio were also calculated. The data analyses were conducted using R 4.0 (R Institute, Vienna, Austria). Approval for the study’s ethics was obtained from the Northern B Health and Disability Ethics Committee, with the reference number 2022 EXP 13741.

## Results

Of the 2837 patients with SLE, 2631 patients had 1 year or more of follow-up data. During the study period (Table 1), hydroxychloroquine was dispensed in 1941/2631 (73.8%) of patients and immunomodulators/biologics were dispensed in 1687/2631 (64.1%). These included azathioprine (1025, 39.0%), mycophenolate mofetil (710, 27.0%), methotrexate (665, 25.3%), cyclophosphamide (194, 7.4%), ciclosporin (142, 5.4%), rituximab (135, 5.1%), leflunomide (109, 4.1%) and tacrolimus (72, 2.7%). Around two thirds (1788/2631, 68.0%) of patients were dispensed with 5 mg or more prednisone daily for at least 90 days, and 605 (23.0%) patients used glucocorticoids at a lower dosage or for a shorter period. Almost all the glucocorticoids dispensed were prednisone (99.6%), and a small proportion was prednisolone (0.2%) and methylprednisolone (0.2%).

**Table 1.** Use of different treatments for SLE by subgroup among patients with ≥1 year follow-up.

Subgroup	All patients	Hydroxychloroquine		Azathioprine		Mycophenolate		Methotrexate		Any immunomodulators/biologics^a^		Glucocorticoid for ≥5 mg daily for ≥90 days	
Gender													
Women	2285	1728	75.6%	904	39.6%	608	26.6%	600	26.3%	1473	64.5%	1555	68.1%
Men	346	213	61.6%	121	35.0%	102	29.5%	65	18.8%	214	61.8%	233	67.3%
Ethnicity													
Asian	398	342	85.9%	177	44.5%	160	40.2%	79	19.8%	293	73.6%	308	77.4%
European/Others	1419	1007	71.0%	525	37.0%	283	19.9%	404	28.5%	845	59.5%	931	65.6%
Māori	412	287	69.7%	152	36.9%	112	27.2%	100	24.3%	272	66.0%	272	66.0%
Pacific	402	305	75.9%	171	42.5%	155	38.6%	82	20.4%	277	68.9%	277	68.9%
Age group													
<20	291	251	86.3%	140	48.1%	123	42.3%	74	25.4%	229	78.7%	223	76.6%
20-29	415	341	82.2%	191	46.0%	171	41.2%	87	21.0%	295	71.1%	305	73.5%
30-39	552	433	78.4%	244	44.2%	177	32.1%	137	24.8%	375	67.9%	370	67.0%
40-49	467	330	70.7%	204	43.7%	123	26.3%	148	31.7%	319	68.3%	330	70.7%
50-59	387	272	70.3%	141	36.4%	69	17.8%	118	30.5%	252	65.1%	261	67.4%
60-69	304	189	62.2%	73	24.0%	34	11.2%	68	22.4%	142	46.7%	185	60.9%
70-79	161	95	59.0%	26	16.1%	13	8.1%	26	16.1%	62	38.5%	90	55.9%
80+	54	30	55.6%	6	11.1%	0	0.0%	7	13.0%	13	24.1%	24	44.4%
Deprivation quintile													
1	386	292	75.6%	181	46.9%	113	29.3%	119	30.8%	265	68.7%	275	71.2%
2	464	343	73.9%	174	37.5%	128	27.6%	121	26.1%	295	63.6%	303	65.3%
3	450	329	73.1%	165	36.7%	113	25.1%	127	28.2%	280	62.2%	308	68.4%
4	605	434	71.7%	225	37.2%	150	24.8%	119	19.7%	365	60.3%	416	68.8%
5	700	538	76.9%	277	39.6%	204	29.1%	179	25.6%	475	67.9%	481	68.7%
Unknown	26	5	19.2%	3	11.5%	2	7.7%	0	0.0%	7	26.9%	5	19.2%
Year of first identification													
Before 2010	1580	1097	69.4%	679	43.0%	330	20.9%	412	26.1%	984	62.3%	1070	67.7%
2010-2012	336	251	74.7%	130	38.7%	101	30.1%	92	27.4%	225	67.0%	235	69.9%
2013-2015	276	224	81.2%	100	36.2%	102	37.0%	74	26.8%	191	69.2%	190	68.8%
2016-2018	250	216	86.4%	74	29.6%	94	37.6%	46	18.4%	158	63.2%	172	68.8%
2019-2021	189	153	81.0%	42	22.2%	83	43.9%	41	21.7%	129	68.3%	121	64.0%
Total	**2631**	**1941**	73.8%	**1025**	39.0%	**710**	27.0%	**665**	25.3%	**1687**	64.1%	**1788**	68.0%

^a^including mycophenolate, methotrexate, leflunomide, azathioprine, cyclophosphamide, tacrolimus, ciclosporin and rituximab.

There were variations in treatment patterns by subgroup. Women were more likely to be dispensed hydroxychloroquine (75.6% vs 61.6%, *p*-value <.001) and methotrexate (26.3% vs 18.8%, *p*-value = .003) than men. Asian patients were more likely to use different treatments than other ethnic groups (all *p*-values <0.05). The proportions of patients using different treatments decreased with age (all *p*-values <0.05): from 86.3%, 78.7% and 76.6% of patients aged less than 20 years old for having hydroxychloroquine, immunomodulators/biologics and glucocorticoid to 55.6%, 24.1% and 44.4% of patients aged 80 years or older, respectively.

During the treatment period of hydroxychloroquine (between the first and last dispensing), 3.1% of patients had an average daily dose of less 50 mg, 6.8% on 50∼100 mg, 28.3% on 100∼200 mg, 30.7% on 200-300 mg, and 31.0% on 300+mg (Appendix Table 1). For azathioprine, 17.5% were on an average daily dose of less than 50 mg, 38.0% on 50∼100 mg, 33.5% on 100∼200 mg, and 11.0% on 200+mg (Appendix Table 2). For mycophenolate, the average daily dose of less than 1000 mg was found in 32.4% of patients, 1000∼2000 mg was in 52.0% of patients, 2000∼3000 mg 13.8% and 3000+mg 1.8% (Appendix Table 3). The average weekly dose of less than 7 mg of methotrexate were seen in 10.5% patients, 7∼14 mg in 35.0% of patients, 14∼21 mg in 28.9% of patients, and 21+mg in 25.6% of patients (Appendix Table 4).

Of the 1941 patients dispensed hydroxychloroquine, 1057/1941 (54.5%) had high adherence (Table 2). There was no difference in adherence to hydroxychloroquine by gender, ethnicity and socioeconomic status (*p*-value = .465, .169, 0.093, respectively). Adherence improved with age (from 47.0% of those <20 years old for having high adherence to 66.7% of patients aged 80+ years, *p*-value = .001) and year of identification (from 48.8% for those identified before 2010 to 73.9% for those in 2019-2021, *p*-value <.001).

**Table 2.** Adherence to hydroxychloroquine by subgroup.

Subgroup	Low adherence		High adherence		Total
Gender					
Women	792	45.8%	936	54.2%	1728
Men	92	43.2%	121	56.8%	213
Ethnicity					
Asian	168	49.1%	174	50.9%	342
European/Others	436	43.3%	571	56.7%	1007
Māori	140	48.8%	147	51.2%	287
Pacific	140	45.9%	165	54.1%	305
Age group					
<20	133	53.0%	118	47.0%	251
20-29	176	51.6%	165	48.4%	341
30-39	201	46.4%	232	53.6%	433
40-49	142	43.0%	188	57.0%	330
50-59	115	42.3%	157	57.7%	272
60-69	66	34.9%	123	65.1%	189
70-79	41	43.2%	54	56.8%	95
80+	10	33.3%	20	66.7%	30
Deprivation quintile					
1	117	40.1%	175	59.9%	292
2	175	51.0%	168	49.0%	343
3	150	45.6%	179	54.4%	329
4	192	44.2%	242	55.8%	434
5	245	45.5%	293	54.5%	538
Unknown	5	100.0%		0.0%	5
Year of first identification					
<2010	562	51.2%	535	48.8%	1097
2010-2012	111	44.2%	140	55.8%	251
2013-2015	102	45.5%	122	54.5%	224
2016-2018	69	31.9%	147	68.1%	216
2019-2021	40	26.1%	113	73.9%	153
Total	**884**	45.5%	**1057**	54.5%	**1941**

The multivariate analysis (Table 3) confirmed most of the treatment pattern found in the descriptive analysis. The odds ratio of dispensing hydroxychloroquine for men compared to women was 0.52 (95% CI: 0.41-0.67), after adjustment for ethnicity, age, socioeconomic status and year of first identification. Asian patients were more likely to use hydroxychloroquine (adjusted odds ratio: 1.68, 95% CI: 1.22-2.33) but were less likely to adhere than Europeans/Others ((adjusted odds ratio: 0.76, 95% CI: 0.59-0.99). In contrast, Māori patients were less likely to use hydroxychloroquine than Europeans/Others (adjusted odds ratio: 0.67, 95% CI: 0.52-0.88). Compared to Europeans/Others, the adjusted odds ratio of dispensing immunomodulators/biologics for Asian patients was 1.36 (95% CI: 1.04-1.77) and the adjusted odds ratio of dispensing prednisone was 1.59 (95% CI: 1.21-2.09).

**Table 3.** Adjusted odds ratio of different treatments for SLE.

Factor	Hydroxychloroquine		Any immunomodulators/biologics^a^		Glucocorticoid for ≥5 mg daily for ≥90 days		High adherence to hydroxychloroquine	
	Adjusted odds ratio (95%CI)	*p*-value	Adjusted odds ratio (95%CI)	*p*-value	Adjusted odds ratio (95%CI)	*p*-value	Adjusted odds ratio (95%CI)	*p*-value
Gender								
Women	Reference		Reference		Reference		Reference	
Men	0.52 (0.41-0.67)	**<0.001**	1.03 (0.80-1.32)	0.805	1.05 (0.82-1.35)	0.713	0.98 (0.71-1.28)	0.873
Ethnicity								
Asian	1.68 (1.22-2.33)	**0.002**	1.36 (1.04-1.77)	**0.023**	1.59 (1.21-2.09)	**0.001**	0.76 (0.59-0.99)	**0.045**
European/Others	Reference		Reference		Reference		Reference	
Māori	0.67 (0.52-0.88)	**0.004**	0.99 (0.77-1.27)	0.911	0.87 (0.67-1.11)	0.261	0.87 (0.66-1.16)	0.351
Pacific	0.83 (0.62-1.12)	0.227	1.08 (0.82-1.41)	0.587	1.00 (0.76-1.31)	1.000	0.95 (0.71-1.28)	0.747
Age group								
<20	Reference		Reference		Reference		Reference	
20-29	0.68 (0.44-1.06)	0.089	0.65 (0.45-0.93)	**0.019**	0.83 (0.58-1.18)	0.295	1.10 (0.78-1.54)	0.598
30-39	0.56 (0.37-0.84)	**0.006**	0.56 (0.40-0.79)	**0.001**	0.59 (0.42-0.82)	**0.002**	1.45 (1.04-2.00)	**0.027**
40-49	0.41 (0.27-0.61)	**<0.001**	0.61 (0.42-0.86)	**0.006**	0.73 (0.51-1.03)	0.073	1.70 (1.20-2.40)	**0.003**
50-59	0.39 (0.26-0.60)	**<0.001**	0.51 (0.36-0.74)	**<0.001**	0.61 (0.43-0.88)	**0.007**	1.74 (1.21-2.49)	**0.003**
60-69	0.28 (0.18-0.44)	**<0.001**	0.24 (0.17-0.36)	**<0.001**	0.46 (0.32-0.67)	**<0.001**	2.24 (1.49-3.37)	**<0.001**
70-79	0.24 (0.15-0.39)	**<0.001**	0.18 (0.11-0.28)	**<0.001**	0.39 (0.25-0.60)	**<0.001**	1.52 (0.92-2.52)	0.095
80+	0.17 (0.09-0.33)	**<0.001**	0.09 (0.04-0.17)	**<0.001**	0.24 (0.13-0.45)	**<0.001**	2.03 (0.88-4.65)	0.873
Deprivation quintile								
1	Reference		Reference		Reference		Reference	
2	0.98 (0.71-1.36)	0.924	0.86 (0.64-1.16)	0.333	0.80 (0.59-1.07)	0.137	0.63 (0.46-0.88)	**0.006**
3	0.92 (0.67-1.27)	0.620	0.77 (0.57-1.04)	0.089	0.91 (0.67-1.23)	0.544	0.77 (0.55-1.07)	0.115
4	0.88 (0.65-1.20)	0.418	0.71 (0.54-0.94)	**0.018**	0.93 (0.70-1.24)	0.619	0.84 (0.61-1.14)	0.258
5	1.14 (0.83-1.57)	0.414	0.90 (0.67-1.20)	0.455	0.91 (0.68-1.22)	0.527	0.81 (0.59-1.10)	0.173
Year of first identification								
Before 2010	Reference		Reference		Reference		Reference	
2010-2012	1.37 (1.04-1.83)	**0.028**	1.26 (0.96-1.63)	0.091	1.13 (0.86-1.47)	0.382	1.37 (1.04-1.82)	**0.028**
2013-2015	1.88 (1.33-2.65)	**<0.001**	1.23 (0.92-1.64)	0.167	0.94 (0.70-1.25)	0.648	1.39 (1.04-1.88)	**0.029**
2016-2018	2.70 (1.81-4.02)	**<0.001**	0.96 (0.71-1.29)	0.780	0.96 (0.71-1.30)	0.798	2.54 (1.84-3.49)	**<0.001**
2019-2021	1.96 (1.30-2.94)	**0.001**	1.28 (0.91-1.81)	0.163	0.79 (0.57-1.10)	0.165	3.32 (2.25-4.90)	**<0.001**

^a^including mycophenolate, methotrexate, leflunomide, azathioprine, cyclophosphamide, tacrolimus, ciclosporin and rituximab.

Bold *p*-values were less than 0.05.

Among 1788 patients using 5 mg or more prednisone daily for at least 90 days, only 581 (32.5%) were dispensed a bisphosphonate (Table 4). For patients over 40 years old, 47.3% had bisphosphonates and this figure was 17.8% for patients under the age of 40 years old. Overall, a smaller proportion of Māori and Pacific patients used bisphosphonates than Asian and Europeans/Others: 24.6% and 14.8% versus 35.7% and 39.0%, respectively (*p*-value <.001). The probability of using bisphosphonates increased with age: from 12.6% of those aged less than 20 years to 75.0% of those aged 80 years or older (*p*-value <.001). Patients with better socioeconomic status had a higher probability of using bisphosphonates than patients with lower socioeconomic status: 35.3%, 38.9% and 36.4% for deprivation quintile 1, 2 and 3 compared to 30.3% and 26.4% for deprivation quintile 4 and 5. Patients (*p*-value <.001). Thirty-eight percent of patients identified before 2010 used bisphosphonates compared to 28.5%, 22.1%, 20.3% and 22.3% patients identified in 2010-2012, 2013-2015, 2016-2018 and 2019-2021, respectively.

**Table 4.** Use of bisphosphonate among patients on glucocorticoid for ≥5 mg daily for ≥90 days.

Subgroup	No bisphosphonate		Had bisphosphonate		Total
Gender					
Women	1046	67.3%	509	32.7%	1555
Men	161	69.1%	72	30.9%	233
Ethnicity					
Asian	198	64.3%	110	35.7%	308
European/Others	568	61.0%	363	39.0%	931
Māori	205	75.4%	67	24.6%	272
Pacific	236	85.2%	41	14.8%	277
Age group					
<20	195	87.4%	28	12.6%	223
20-29	252	82.6%	53	17.4%	305
30-39	291	78.6%	79	21.4%	370
40-49	223	67.6%	107	32.4%	330
50-59	139	53.3%	122	46.7%	261
60-69	70	37.8%	115	62.2%	185
70-79	31	34.4%	59	65.6%	90
80+	6	25.0%	18	75.0%	24
Deprivation quintile					
1	178	64.7%	97	35.3%	275
2	185	61.1%	118	38.9%	303
3	196	63.6%	112	36.4%	308
4	290	69.7%	126	30.3%	416
5	354	73.6%	127	26.4%	481
Unknown	4	80.0%	1	20.0%	5
Year of first identification					
Before 2010	660	61.7%	410	38.3%	1070
2010-2012	168	71.5%	67	28.5%	235
2013-2015	148	77.9%	42	22.1%	190
2016-2018	137	79.7%	35	20.3%	172
2019-2021	94	77.7%	27	22.3%	121
Total	**1207**	67.5%	**581**	32.5%	**1788**

After adjustment for ethnicity, age, socioeconomic status and year of identification, men were less likely to be dispensed a bisphosphonate (adjusted odds ratio: 0.68, 95% CI: 0.49-0.95, Table 5). Compared to Europeans/Others, the adjusted odds ratio of using bisphosphonates was 1.91 (95% CI: 1.40-2.61) for Asian and 0.55 (95% CI: 0.37-0.83) for Pacific patients. After adjustment for other factors, men had a lower probability of using bisphosphonates (adjusted odds ratio: 0.68, 95% CI: 0.49-0.95). Compared to patients aged less than 20 years old, the adjusted odds ratio of using bisphosphonates increased with age, from 1.35 for those aged 20-29 years to 23.76 for those aged 80 years or older.

**Table 5.** Adjusted odds ratio of having bisphosphonate among patients on glucocorticoid for ≥5 mg daily for ≥90 days.

Factor	Adjusted odds ratio (95%CI)	*p*-value
Gender		
Women	Reference	
Men	0.68 (0.49-0.95)	**0.024**
Ethnicity		
Asian	1.91 (1.40-2.61)	**<0.001**
European/Others	Reference	
Māori	0.84 (0.60-1.19)	0.335
Pacific	0.55 (0.37-0.83)	**0.004**
Age group		
<20	Reference	
20-29	1.35 (0.81-2.24)	0.247
30-39	1.63 (1.01-2.64)	**0.045**
40-49	2.88 (1.80-4.63)	**<0.001**
50-59	5.55 (3.42-9.01)	**<0.001**
60-69	11.86 (7.01-20.06)	**<0.001**
70-79	13.30 (7.13-24.79)	**<0.001**
80+	23.76 (8.46-66.74)	**<0.001**
Deprivation quintile		
1	Reference	
2	0.90 (0.62-1.30)	0.573
3	1.00 (0.69-1.45)	0.995
4	0.67 (0.47-0.96)	**0.029**
5	0.83 (0.58-1.20)	0.329
Year of first identification		
Before 2010	Reference	
2010-2012	0.59 (0.42-0.84)	**0.003**
2013-2015	0.46 (0.31-0.69)	**<0.001**
2016-2018	0.43 (0.28-0.67)	**<0.001**
2019-2021	0.41 (0.25-0.67)	**<0.001**

Bold *p*-values were less than 0.05.

## Discussion

This is the first study to report on treatment patterns of SLE in Aotearoa/New Zealand. We know from recent epidemiological studies that SLE has a significant presence in our country.^14,15^ This study found that there were disparities in treatment of SLE by subgroup; adherence to hydroxychloroquine was suboptimal; and the majority of patients were taking prolonged glucocorticoids, with less than half of patients over the age of 40 years using bisphosphonates.

EULAR guidelines recommend hydroxychloroquine for all patients with SLE, given its benefits of preventing flares of SLE and increasing long term survival for patients.^1,3^ Our study notes 73.8% of patients with SLE were dispensed hydroxychloroquine, whilst this is lower than the guidelines recommend, similar rates are seen in other countries: 69.5% in the UK,^17^ 72.9% in South Korea,^18^ 75% in the Asia-Pacific Collaboration cohort^19^ and 85% in Australia.^20^ We also found that 64.1% of patients have ever used immunomodulators/biologics, which is similar to what was found in an Asia-Pacific Collaboration cohort (68.9%).^19^ Our study shows that common immunomodulators include azathioprine in 39.0% of SLE patients, mycophenolate in 27.0% and methotrexate in 25.3% of patients. These three medications are supported by guidelines for use in moderate or severe SLE and cover most organ manifestations.^2^ These rates of use are higher than those seen nearby in Australia with 13% of SLE patients using azathioprine, 11.5% mycophenolate and 28% methotrexate.^20^ The reasons for this discrepancy are unclear. Interestingly, mycophenolate mofetil was only publicly funded for non-renal SLE from 2014. Taiwan also seems to predominantly use azathioprine 28% over mycophenolate 4.8% and methotrexate 4.5%.^21^

Internationally, biologic medications are playing a bigger role in the treatment of SLE. The EULAR guidelines note that belimumab (an inhibitor of B-lymphocyte stimulator (BLys)) can be used in extra-renal disease if there is inadequate control despite first line treatment.^2^ Rituximab is an anti-CD20 monoclonal antibody that depletes B lymphocytes and, despite negative results of randomised controlled trials, can be used off label in patients with severe renal or extra-renal disease refractory to other agents.^2^ Rituximab became publicly funded in New Zealand for use in SLE in 2014 but belimumab is not yet available. Despite restrictions on prescribing and a requirement to only use rituximab if other lines of therapy (i.e., cyclophosphamide) are inappropriate, 5.1% of SLE patients use rituximab in addition to 7.4% for cyclophosphamide. These rates are also higher than in Australia which report 0.4% of patients using cyclophosphamide and 0.9% rituximab.^20^

We found that the probability of having hydroxychloroquine, immunomodulators/biologics and glucocorticoids decreased with age. Similar results were found in South Korea, showing 85.5% of patients aged less than 50 years at diagnosis received hydroxychloroquine compared to 67.7% of aged 50 years or older at diagnosis.^18^ The exact reasons remain unclear but are probably due to patient or physician concerns regarding retinopathy risk and long-term hydroxychloroquine exposure.^18^ Alternatively, those with more severe disease may have died at a younger age leaving a cohort with milder disease not requiring immunosuppressive therapies. There is also some evidence that SLE disease activity becomes less pronounced with advancing age,^22^ negating the need for medication in the elderly population.

Men were less likely to take hydroxychloroquine which is concerning. It is not possible to ascertain from these data sources if the medication was prescribed but not collected, or simply not prescribed. Discrepancies between outcomes and clinical characteristics have been noted between men and women with SLE.^23^ Previous studies have noted lower levels of health literacy in male patients with SLE compared to women^24^ as well as higher age at disease onset and diagnosis with more late-onset SLE.^25^ Multiple studies show that male sex is associated with damage accrual.^7,25,26^ Men with SLE also appear to have more coronary artery disease and myocardial infarctions^23^ which may relate to the general increased cardiovascular risk in men compared to women.^27^ Nonetheless, it appears that men with SLE in Aotearoa New Zealand are missing out on the mortality benefits of hydroxychloroquine.

Asians were more likely to receive hydroxychloroquine, immunomodulators/biologics and glucocorticoids, but Māori were less likely to have hydroxychloroquine. Recent epidemiology research shows that Māori patients had twice the incidence and prevalence of Europeans.^15^ This research has also shown that Māori patients were 15 years younger and Pacific patients were 20 years younger than Europeans/others at the age of death.^14^ Māori, Pacific and Asian patients were more likely to have SLE as the underlying cause of death and after adjustment for age, Māori were more likely to die than Europeans for all-cause mortality (HR 1.72) and SLE specific mortality (HR 2.60).^14^ This patient group is most likely to benefit from hydroxychloroquine therapy and the reasons for reduced use are unclear.

Adherence to hydroxychloroquine is a common issue and barriers to adherence include concern with adverse effects, perceived inefficacy, non-specific symptoms attributed to hydroxychloroquine, forgetfulness and failure to accept the illness and benefits of therapy.^1,2,28^ Low adherence to hydroxychloroquine can lead to worse SLE outcomes. It has been reported that low adherence to hydroxychloroquine was associated with six times higher risk of severe flare, 45% higher probability of hospitalisation and eight times higher risk of death.^29–32^ In New Zealand, 45.5% of patients had low adherence to hydroxychloroquine; that is higher than the rate in France (38%),^4^ but was lower than the percentage in the US (85%).^6^ There were differences in adherence rate by ethnicity, age group and socioeconomic status in New Zealand, which have been found in other countries as well. African descendants, younger patients, and patients with lower socioeconomic status were more likely to not adhere to hydroxychloroquine treatment.^6,29^ Other factors were also reported to increase the risk of non-adherence, including increased SLE-related comorbidities and lower hospital concentration.^6,29^

Glucocorticoids are commonly used in the management of SLE flares with current guidelines supporting their use for the rapid relief of symptoms, yet recommending minimising use and cessation if possible.^1^ Evidence suggests that glucocorticoids may contribute to organ damage accrual in SLE, independent of the presence of clinical or serological disease activity^33^ with higher cumulative doses being associated with greater damage accrual.^34,35^ The Hopkins study demonstrated that a 1 mg/day increase in prednisone dose was associated with a 2.8% increase in the risk of developing new organ damage.^36^ Aside from damage accrual, prednisone at doses ≥7.5 mg/day increase the risk of osteoporosis with a hazard ratio of 2.16, cataracts (hazard ratio 2.41) and cardiovascular damage (hazard ratio 1.44).^36^

The American College of Rheumatology Guidelines for glucocorticoid-induced osteoporosis prevention and treatment separate patients into those above or below 40 years of age.^37^ Bisphosphonates are recommended for anyone with a moderate to high risk of an osteoporotic fracture, which often takes into consideration past fracture history, bone mineral density T score (or Z score if less than 40 years), fracture risk using a risk calculator (i.e. Fracture Risk Assessment Tool (FRAX)) and use of very high doses of glucocorticoids.^37^ The threshold to treat is generally lower for patients older than 40 years. Our data shows that glucocorticoid use is not supplemented with bisphosphonate use in 52.7% of those aged over 40 years old and on long-term prednisone, and in 82.2% of those aged under 40 years old. However, we did not have data on the fracture risk to assess whether bisphosphonates were sufficiently prescribed in patients in need.

In New Zealand, 68% of SLE patients were dispensed glucocorticoid for at least 5 mg daily for 90 days or longer. This is much higher than the rate in Australia where 43.1% of SLE patients had ever received glucocorticoid,^20^ and is higher than the rate in the UK (51.9%).^17^ To reduce glucocorticoid exposure to patients, the EULAR Guidelines recommend the addition of alternative immunomodulators,^1,38^ which can facilitate rapid glucocorticoid weaning and help prevent disease flares.^8^ The choice of immunomodulators depends on the disease manifestation, patient age, childbearing potential, safety and cost. Organ threatening disease is traditionally treated with cyclophosphamide, which, whilst efficacious in treatment, can be associated with its own organ damage accrual.^8^ One key aspect to facilitate this study is to improve the funded pharmacological options for management of SLE in New Zealand.

The strengths of this study included very detailed data on medication dispensing that allowed us to examine the usage of different treatments for SLE and the adherence. We have compared the usage of different treatments for SLE by gender, age, ethnicity and socioeconomic status, which have rarely been reported internationally. This study is not exempt from limitations. All the SLE cases were identified through healthcare administrative datasets. The SLE diagnosis was made by clinicians, but we could not confirm that the diagnosis was met by the ACR/EULAR, SLICC or ACR classification criteria. The study timeframe would span the evolution of SLE criteria from ACR through SLICC etc and application of a single set of criteria would not be sensible. This cohort were seen in rheumatology and renal clinics and as inpatients over a period of time and we are not relying on a single instance of SLE diagnosis. The use of medications and adherence to hydroxychloroquine were based on pharmaceutical dispensing data from pharmacists, therefore there is uncertainty regarding whether the medications dispensed were taken by patients. The use of medications and adherence to hydroxychloroquine might be slightly overestimated in this study. We did not have data on disease severity nor reason for cessation of treatment. Some patients who were classified as having low adherence to hydroxychloroquine could be due to side effects, remission or moving overseas. Patients in some subgroups were more likely to receive treatment, and this was probably because they had more severe disease, but we do not have data to explore the reasons.

## Conclusion

Hydroxychloroquine is the first line treatment for most patients treated for SLE. Adherence to hydroxychloroquine in these patients varied and was lower in men and Māori. There were higher rates of prescription of immunomodulators compared to other countries. Azathioprine, mycophenolate and methotrexate were the most commonly used immunomodulators. Prednisone is commonly prescribed and used long term. Half of those over the age of 40 years old co-administered bisphosphonate. The use of SLE treatments decreased with age, but bisphosphonate usage and adherence to hydroxychloroquine improved with age. Further research is needed to identify the reasons to these discrepancies on SLE treatments by gender, ethnicity, age and socioeconomic status.

## Appendix

**Appendix Table 1.** Average daily dose of hydroxychloroquine between first and last dispensing.

Subgroup	<50 mg		50∼100 mg		100∼200 mg		200-300 mg		300+ mg		Total
Gender											
Women	56	3.2%	121	7.0%	499	28.9%	528	30.6%	524	30.3%	1728
Men	5	2.3%	11	5.2%	51	23.9%	68	31.9%	78	36.6%	213
Ethnicity											
Asian	8	2.3%	19	5.6%	106	31.0%	123	36.0%	86	25.1%	342
European/Others	31	3.1%	55	5.5%	264	26.2%	309	30.7%	348	34.6%	1007
Māori	11	3.8%	31	10.8%	84	29.3%	82	28.6%	79	27.5%	287
Pacific	11	3.6%	27	8.9%	96	31.5%	82	26.9%	89	29.2%	305
Age group											
<20	11	4.4%	31	12.4%	91	36.3%	74	29.5%	44	17.5%	251
20-29	9	2.6%	33	9.7%	108	31.7%	93	27.3%	98	28.7%	341
30-39	16	3.7%	28	6.5%	135	31.2%	128	29.6%	126	29.1%	433
40-49	14	4.2%	24	7.3%	78	23.6%	111	33.6%	103	31.2%	330
50-59	7	2.6%	8	2.9%	72	26.5%	80	29.4%	105	38.6%	272
60-69	3	1.6%	3	1.6%	41	21.7%	58	30.7%	84	44.4%	189
70-79	1	1.1%	3	3.2%	22	23.2%	33	34.7%	36	37.9%	95
80+	0	0.0%	2	6.7%	3	10.0%	19	63.3%	6	20.0%	30
Deprivation quintile											
1	8	2.7%	17	5.8%	75	25.7%	107	36.6%	85	29.1%	292
2	11	3.2%	20	5.8%	106	30.9%	107	31.2%	99	28.9%	343
3	10	3.0%	26	7.9%	83	25.2%	106	32.2%	104	31.6%	329
4	14	3.2%	24	5.5%	123	28.3%	134	30.9%	139	32.0%	434
5	18	3.3%	44	8.2%	162	30.1%	141	26.2%	173	32.2%	538
Unknown			1		1		1		2		5
Year of first identification											
Before 2010	40	3.6%	359	32.7%	325	29.6%	287	26.2%	86	7.8%	1097
2010-2012	6	2.4%	64	25.5%	80	31.9%	81	32.3%	20	8.0%	251
2013-2015	7	3.1%	63	28.1%	65	29.0%	75	33.5%	14	6.3%	224
2016-2018	5	2.3%	39	18.1%	83	38.4%	81	37.5%	8	3.7%	216
2019-2021	3	2.0%	25	16.3%	43	28.1%	78	51.0%	4	2.6%	153
Total	**61**	3.1%	**132**	6.8%	**550**	28.3%	**596**	30.7%	**602**	31.0%	**1941**

**Appendix Table 2.** Average daily dose of azathioprine between first and last dispensing.

Subgroup	<50 mg		50∼100 mg		100∼200 mg		200+ mg		Total
Gender									
Women	158	17.5%	343	37.9%	302	33.4%	101	11.2%	904
Men	21	17.4%	47	38.8%	41	33.9%	12	9.9%	121
Ethnicity									
Asian	39	22.0%	72	40.7%	50	28.2%	16	9.0%	177
European/Others	74	14.1%	189	36.0%	194	37.0%	68	13.0%	525
Māori	34	22.4%	55	36.2%	43	28.3%	20	13.2%	152
Pacific	32	18.7%	74	43.3%	56	32.7%	9	5.3%	171
Age group									
<20	37	26.4%	63	45.0%	29	20.7%	11	7.9%	140
20-29	41	21.5%	75	39.3%	53	27.7%	22	11.5%	191
30-39	37	15.2%	99	40.6%	80	32.8%	28	11.5%	244
40-49	28	13.7%	67	32.8%	89	43.6%	20	9.8%	204
50-59	19	13.5%	51	36.2%	51	36.2%	20	14.2%	141
60-69	9	12.3%	24	32.9%	31	42.5%	9	12.3%	73
70-79	6	23.1%	9	34.6%	8	30.8%	3	11.5%	26
80+	2	33.3%	2	33.3%	2	33.3%		0.0%	6
Deprivation quintile									
1	31	17.1%	51	28.2%	75	41.4%	24	13.3%	181
2	29	16.7%	67	38.5%	60	34.5%	18	10.3%	174
3	27	16.4%	65	39.4%	55	33.3%	18	10.9%	165
4	34	15.1%	98	43.6%	70	31.1%	23	10.2%	225
5	57	20.6%	108	39.0%	82	29.6%	30	10.8%	277
Unknown	1		1		1				3
Year of first identification									
Before 2010	122	18.0%	273	40.2%	214	31.5%	70	10.3%	679
2010-2012	26	20.0%	41	31.5%	47	36.2%	16	12.3%	130
2013-2015	16	16.0%	36	36.0%	35	35.0%	13	13.0%	100
2016-2018	9	12.2%	27	36.5%	27	36.5%	11	14.9%	74
2019-2021	6	14.3%	13	31.0%	20	47.6%	3	7.1%	42
Total	**179**	17.5%	**390**	38.0%	**343**	33.5%	**113**	11.0%	**1025**

**Appendix Table 3.** Average daily dose of mycophenolate between first and last dispensing.

Subgroup	<1000 mg		1000∼2000 mg		2000∼3000 mg		3000+ mg		Total
Gender									
Women	202	33.2%	312	51.3%	84	13.8%	10	1.6%	608
Men	28	27.5%	57	55.9%	14	13.7%	3	2.9%	102
Ethnicity									
Asian	43	26.9%	92	57.5%	23	14.4%	2	1.3%	160
European/Others	81	28.6%	160	56.5%	38	13.4%	4	1.4%	283
Māori	46	41.1%	50	44.6%	15	13.4%	1	0.9%	112
Pacific	60	38.7%	67	43.2%	22	14.2%	6	3.9%	155
Age group									
<20	49	39.8%	59	48.0%	14	11.4%	1	0.8%	123
20-29	53	31.0%	89	52.0%	23	13.5%	6	3.5%	171
30-39	63	35.6%	85	48.0%	26	14.7%	3	1.7%	177
40-49	28	22.8%	73	59.3%	21	17.1%	1	0.8%	123
50-59	19	27.5%	39	56.5%	9	13.0%	2	2.9%	69
60-69	12	35.3%	19	55.9%	3	8.8%		0.0%	34
70-79	6	46.2%	5	38.5%	2	15.4%		0.0%	13
80+									
Deprivation quintile									
1	38	33.6%	54	47.8%	19	16.8%	2	1.8%	113
2	37	28.9%	76	59.4%	15	11.7%		0.0%	128
3	30	26.5%	62	54.9%	17	15.0%	4	3.5%	113
4	47	31.3%	82	54.7%	18	12.0%	3	2.0%	150
5	77	37.7%	95	46.6%	28	13.7%	4	2.0%	204
Unknown	1				1				2
Year of first identification									
Before 2010	117	35.5%	184	55.8%	25	7.6%	4	1.2%	330
2010-2012	36	35.6%	50	49.5%	14	13.9%	1	1.0%	101
2013-2015	28	27.5%	57	55.9%	16	15.7%	1	1.0%	102
2016-2018	29	30.9%	43	45.7%	18	19.1%	4	4.3%	94
2019-2021	20	24.1%	35	42.2%	25	30.1%	3	3.6%	83
Total	**230**	32.4%	**369**	52.0%	**98**	13.8%	**13**	1.8%	**710**

**Appendix Table 4.** Average weekly dose of methotrexate between first and last dispensing.

Subgroup	<7 mg		7∼14 mg		14∼21 mg		21+ mg		Total
Gender									
Women	64	10.7%	206	34.3%	178	29.7%	152	25.3%	600
Men	6	9.2%	27	41.5%	14	21.5%	18	27.7%	65
Ethnicity									
Asian	11	13.9%	31	39.2%	16	20.3%	21	26.6%	79
European/Others	33	8.2%	131	32.4%	132	32.7%	108	26.7%	404
Māori	14	14.0%	40	40.0%	24	24.0%	22	22.0%	100
Pacific	12	14.6%	31	37.8%	20	24.4%	19	23.2%	82
Age group									
<20	8	10.8%	25	33.8%	20	27.0%	21	28.4%	74
20-29	12	13.8%	27	31.0%	25	28.7%	23	26.4%	87
30-39	18	13.1%	35	25.5%	49	35.8%	35	25.5%	137
40-49	13	8.8%	57	38.5%	41	27.7%	37	25.0%	148
50-59	10	8.5%	43	36.4%	29	24.6%	36	30.5%	118
60-69	4	5.9%	30	44.1%	22	32.4%	12	17.6%	68
70-79	4	15.4%	11	42.3%	6	23.1%	5	19.2%	26
80+	1	14.3%	5	71.4%		0.0%	1	14.3%	7
Deprivation quintile									
1	8	6.7%	46	38.7%	35	29.4%	30	25.2%	119
2	16	13.2%	37	30.6%	33	27.3%	35	28.9%	121
3	13	10.2%	41	32.3%	44	34.6%	29	22.8%	127
4	14	11.8%	39	32.8%	36	30.3%	30	25.2%	119
5	19	10.6%	70	39.1%	44	24.6%	46	25.7%	179
Unknown									
Year of first identification									
Before 2010	47	11.4%	156	37.9%	119	28.9%	90	21.8%	412
2010-2012	14	15.2%	29	31.5%	30	32.6%	19	20.7%	92
2013-2015	4	5.4%	24	32.4%	20	27.0%	26	35.1%	74
2016-2018	2	4.3%	11	23.9%	15	32.6%	18	39.1%	46
2019-2021	3	7.3%	13	31.7%	8	19.5%	17	41.5%	41
Total	**70**	10.5%	**233**	35.0%	**192**	28.9%	**170**	25.6%	**665**
